# Correspondence

**Published:** 1897-11

**Authors:** A. B. Reeves

**Affiliations:** Kendalia, Texas


### Correspondence.

KENDALIA, TEXAS, September, 1897.

*Editors Texas Medical Journal:*

It has falllen to me to meet a case of what is generally supposed to be very rare, a case of "Hodgkin's Disease," so called. According to the description given in Flint's Practice (edition of 1881) this is a typical case.

A man, 36 years of age, of a scrofulous diathesis: The first abnormality noticed, some sixteen months ago, was an enlargement of the cervical glands. The enlargement was gradual, and later those of the axilla and groin. He was seen by several physicians, but no diagnosis was made. They and other friends, however, advised him to place himself in the hands of a physician for treatment, but he steadily declined to do so for some time, saying that he suffered no pain or inconvenience whatever. Except for the deformity and unsightliness, he was apparently a strong, healthy man in perfect health; he followed daily his business, that of a stockraiser and overseer of the public roads. This he continued to do up to about ten months ago, when, at the request of himself and relatives, I took charge of the case. I read up carefully on diseases of the glandular system, and soon decided that I had to deal with a case of Hodgkin's disease. I prescribed iodide potassium, bichloride mercury, phytolacca, etc., running the range of alteratives, using externally tincture of iodine at the same time. No improvement was noticeable.

Seeing his condition was no better after my efforts, I suggested consultation, in the hope of getting the light of experience on this ease, for I had never seen one like it before. Accordingly the patient was seen by Drs. Braunnagel, Bleim, Graves and several other physicians of San Antonio. Neither of them, however, had anything to offer, and gave him no encouragement. As he was under my immediate charge, I had daily opportunity of observing the progress of the case. There was nothing noticeable, as stated, but a gradual increase in the swelling in the lymphatic glands, particularly in the axillary and inquinal regions. Presently the spleen began to show involvement. It became so congested and enlarged as to seriously interfere with respiration. I made, of course, an unfavorable prognosis, and as he has a large family dependent on him, suggested that he put his affairs in shape for the inevitable. His wife, as a last resort, asked that Dr. Miller, of Boerne, be requested to see him. Dr. Miller came, and made a searching inquiry into the history and progress of the case. He gave it as his belief that Hodgkin's disease is in no way different from scrofulous tubercular diseases of the lymphatic system. Examination of the most recent literature at my command verifies the doctor's opinion, which was also confirmed by post mortem examination. It was a case of tubercular affection of the lymphatics. I agree with Dr. Miller, that it is hair-splitting to designate known diseases by the title of individuals who point out distinctions where there are no differences. It confuses rather than enlightens the reader.

A. B. Reeves, M. D.

